# Commentary: Acanthocytes identified in Huntington's disease

**DOI:** 10.3389/fnins.2022.1049676

**Published:** 2022-11-04

**Authors:** Kevin Peikert, Alexander Storch, Andreas Hermann, G. Bernhard Landwehrmeyer, Ruth H. Walker, Greta Simionato, Lars Kaestner, Adrian Danek

**Affiliations:** ^1^Translational Neurodegeneration Section “Albrecht Kossel”, Department of Neurology, University Medical Center Rostock, University of Rostock, Rostock, Germany; ^2^Department of Neurology, University Medical Center Rostock, University of Rostock, Rostock, Germany; ^3^Center for Transdisciplinary Neurosciences Rostock (CTNR), University Medical Center Rostock, University of Rostock, Rostock, Germany; ^4^DZNE, Deutsches Zentrum für Neurodegenerative Erkrankungen, German Center for Neurodegenerative Diseases, Research Site Rostock/Greifswald, Rostock, Germany; ^5^Department of Neurology, University of Ulm, Ulm, Germany; ^6^Department of Neurology, James J. Peters Veterans Affairs Medical Center, Bronx, NY, United States; ^7^Department of Neurology, Mount Sinai School of Medicine, New York, NY, United States; ^8^Experimental Physics, Saarland University, Saarbruecken, Germany; ^9^Institute for Clinical and Experimental Surgery, Saarland University, Campus University Hospital, Homburg, Germany; ^10^Theoretical Medicine and Biosciences, Saarland University, Homburg, Germany; ^11^Neurologische Klinik und Poliklinik, Ludwig-Maximilians-Universität München, Munich, Germany; ^12^DZNE, Deutsches Zentrum für Neurodegenerative Erkrankungen, German Center for Neurodegenerative Diseases, Munich, Germany

**Keywords:** acanthocytosis, huntingtonism, biomarkers, neurodegeneration, neuroacanthocytosis, movement disorders, erythrocyte classification, differential diagnosis

Using scanning electron microscopy (SEM), Yu et al. described a significant percentage of acanthocytes in peripheral blood from a subgroup of patients (four out of 40) diagnosed with Huntington's Disease (HD) (Yu et al., 2022). Applying whole-exome sequencing as well as clinical reasoning the authors excluded both genetic and other causes for their finding of elevated acanthocyte numbers. The authors conclude that their findings highlight the complexity and diversity of HD.

Increased acanthocytes have been identified as a useful diagnostic finding in conditions with huntingtonism (Feigin and Talbot, 2014) that in the past were grouped under “neuroacanthocytosis syndromes,” an umbrella term to be employed with greatest care (Walker and Danek, 2021). We have concerns that Yu et al.'s report will add to the pre-existing confusion that we have been attempting for several years to dispel regarding diagnosis and classification of these disorders.

Introduced by Karl Singer, acanthocytes, defined by their “thorny” appearance (Greek *akantha* = thorn), “show several, irregularly spaced, relatively large, coarse, spiculate projections from the surface of the cell, which vary in length and width.” This appearance was felt to be clearly different from the shape of “normal erythrocytes, becoming crenated by exposure to hypertonic salt solutions” (Singer et al., 1952). The term, “echinocyte” (Greek *echinos* = sea urchin), was introduced to denote “the crenated, spiculated form” of normal erythrocytes “produced by alterations in the intra- or extracellular environment.” The acanthocytes of abetalipoproteinemia (previously denoted as a “neuroacanthocytosis” disorder), in contrast, on SEM were found to “display four to eight large, smooth surface projections from an irregular central mass.” (Bessis and Lessin, 1970). The additional terms “spur cells” (Smith et al., 1964) and “burr cells” (likened “to the prickly envelope of a burr”; Schwartz and Motto, 1949), were declared redundant (Brecher and Bessis, 1972). Normal erythrocytes exposed to appropriate stress to induce echinocyte formation were classified into three stages: echinocytes type I—irregularly contoured disks; II—flat cells with spicules; III—ovoid or spherical cells with 10–30 spicules evenly distributed over the surface (Brecher and Bessis, 1972). Once SEM became the standard for classification (Bessis et al., 1973), Redman et al. further proposed to classify acanthocytosis-prone erythrocytes (as seen in the McLeod phenotype) in five stages based on the shapes they assumed under native and experimental conditions: I—normal discocyte; II—stomatocyte with cuplike invagination; III stomatocyte with many smaller, deep invaginations; AI—acanthocytes I with irregular surfaces; AII—ovoid acanthocytes, with several projections or spicules (Redman et al., 1989).

The International Council for Standardization in Hematology currently makes recommendations for visual classification of stained blood films (Palmer et al., 2015), which are, however, less precise in comparison to the above discussions based on SEM. There is a trend to facilitate diagnosis by automation (Kamentsky, 1973) with the goal of obviating these often tricky and time-consuming classificatory decisions by human observers. We recently proposed a protocol for confocal imaging with artificial neural networks that had been trained on six classes of abnormal erythrocytes (acanthocytes included) and a continuous scale of erythrocyte shape transitions (discocyte to echinocyte, or to spherocyte *via* stomatocyte, respectively; see Figure 1; Simionato et al., 2021).

**Figure 1:**
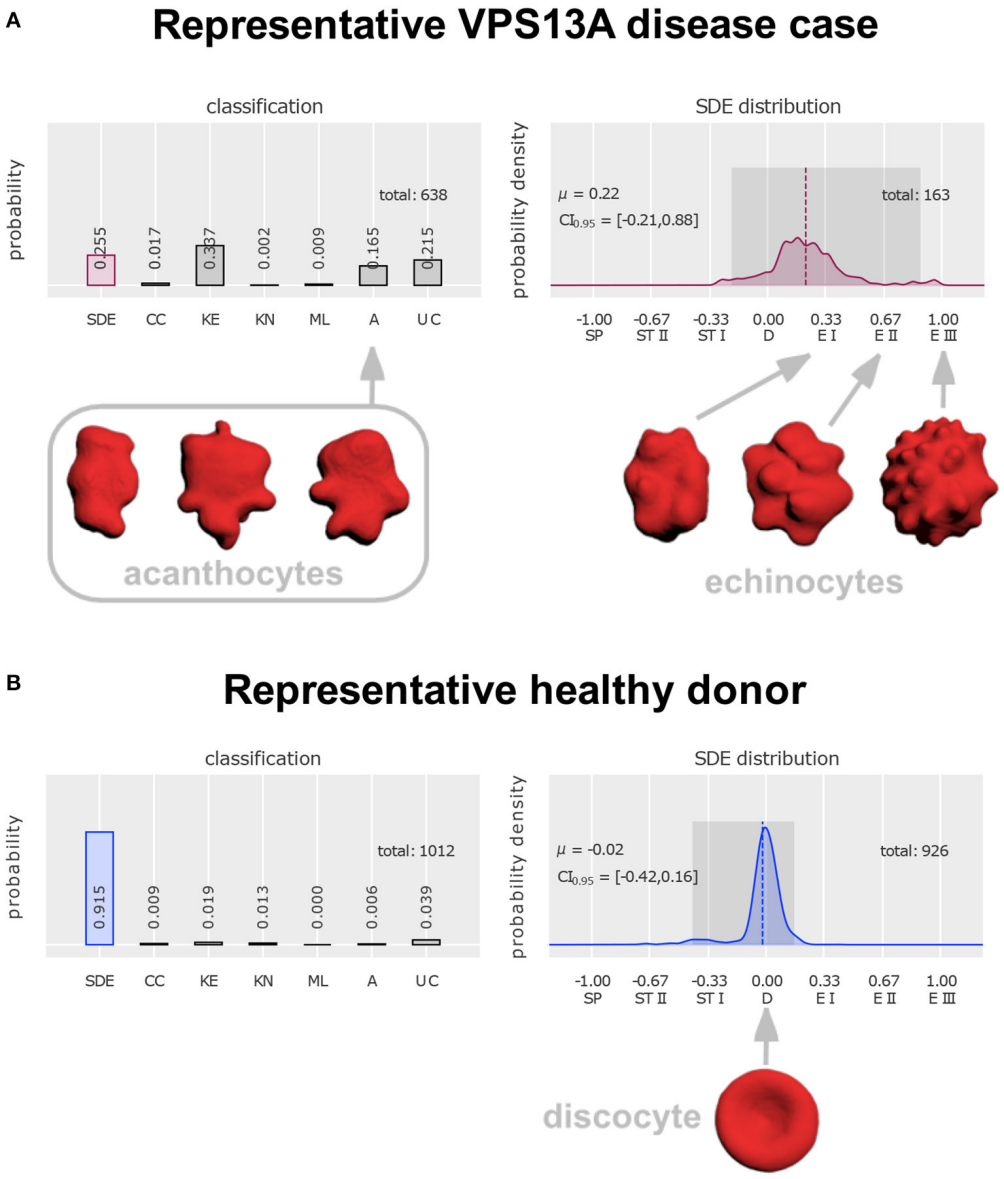
Distinction of acanthocytes from echinocytes based on 3D confocal images and classification with artificial neural networks [figure modified from Rabe et al. (2021)]. To arrive at these classifications, a drop of blood was dropped directly (i.e., without anticoagulant exposure) from the blood-drawing needle tip into glutaraldehyde for fixation (Abay et al., 2019), followed by staining with Cell Mask deep red and confocal microscopic imaging (Quint et al., 2017). Artificial intelligence-based classification allows unbiased automated analysis, including a “stomatocyte-discocyte-echinocyte” (SDE) classification on a continuous scale (instead of in discrete classes). For design and validation of the artificial neural network see (Simionato et al., 2021). **(A)** Classification of erythrocytes from a patient with genetically proven VPS13A disease (chorea-acanthocytosis). The first column on the left reflects the fraction of cells that follow the SDE sequence that erythrocytes show under appropriate environmental conditions. Their distribution is plotted in the right diagram. Its zero value denotes perfect discocytes (D) and positive or negative deviations correspond to echinocytosis (E I–E III) and spherocytosis (SP)/stomatocytosis (ST I–ST II), respectively. Examples of echinocyte types I to III are depicted on the right. The left diagram represents abnormal cell shapes, namely cell clusters (CC), keratocytes (KE), knizocytes (KN), multilobate cells (ML), acanthocytes (A), and unclassifiable cells (UC). Examples of acanthocytes are depicted on the left. **(B)** Erythrocyte classification of a representative healthy donor and depiction of a perfect discocyte. For patient **(A)** and control a total of 638 and 1,012 erythrocytes, respectively, were analyzed. In the control a majority of 91.5% conformed with the SDE range (only 25.5% in the patient). There were 16.5% acanthocytes in the patient sample. Apart from detection of a few acanthocytes (0.6%) also in the control, the patient displayed various other irregularly shaped cells (keratocytes, knizocytes, multilobate cells, and unclassifiable cells) in greater abundance (59.3%) than the control (7%). Further, the probability density of the patient's SDE distribution and that of the control differ considerably—due to the presence of echinocytes in the patient that are almost completely absent in the control.

In their SEM study Yu et al. defined acanthocytes as contracted erythrocytes “with a number of irregularly spaced thorny surface projections” and considered them abnormal if their occurrence exceeded 3%. The blood samples had been freshly fixed in 2.5% glutaraldehyde and 4% paraformaldehyde and processed for SEM, the resultant images being analyzed by a board-certified pathologist. Yu et al. concluded that the spectrum of HD may include red blood cell acanthocytosis. The occurrence of acanthocytes outside the limit of normal has so far been thought to be incompatible with a diagnosis of HD and must therefore be interpreted with caution. Three aspects of their work ought to be reflected in particular: (1) difficulties in classification of misshapen erythrocytes; (2) consideration of previous work; (3) role of acanthocyte testing for clinical diagnosis in general.

As already mentioned, the distinction of acanthocytes from other shapes of erythrocytes can be difficult as their definitions may appear insufficient in the individual case. We wonder why Yu et al. relied on the expertise of only one single rater. In contrast, four experienced raters (LK, GS, AS, RHW) in unison identified the majority of non-discocytes in the SEM images of Yu et al. as echinocytes (stages E I or E II, see Figure 1 below). The spicules appeared too small and too regular to classify these cells as acanthocytes. Upon application of our cell shape classification method with artificial neural networks the existence of clear differences between acanthocytes and echinocytes was confirmed (Figure 1; Rabe et al., 2021). The main advantage of this approach is that “prototypes” (e.g., acanthocytes vs. echinocytes) are used for training of the network, which extracts main features and applies criteria for shape classification independent from individual raters.

Earlier studies of erythrocyte morphology in HD failed to detect acanthocytes (Butterfield et al., 1977; Markesbery and Butterfield, 1977; Zanella et al., 1980; Dubbelman et al., 1981; McCormack et al., 1982; Beverstock, 1984; Sassone et al., 2009). Most recently, this was the case in 13 HD patients in whom Storch et al.'s standard technique was applied (Anderson et al., 2017). The systematic prospective reader-blinded analysis of acanthocytosis in movement disorders by Storch et al. is still the *de facto* standard (albeit without EM confirmation; Storch et al., 2005). Their protocol recommends using isotonically diluted blood samples as unfixed wet preparation and analysis with phase contrast microscopy. The higher sensitivity than that of previous protocols results from the higher susceptibility of acanthocytes to echinocytic stress (here isotonic dilution) in comparison to erythrocytes from healthy donors. According to this protocol, abnormally shaped cells must not exceed a proportion of 6.3% of total erythrocytes (specificity 0.98; sensitivity 1.0). As phase contrast microscopy cannot easily distinguish acanthocytes from echinocytes, all erythrocytes “with spicules, which were irregular in shape and orientation/distribution (corresponding to type AI/AII acanthocytes and echinocytes in Redman's classification using SEM)” were classified as “abnormal.” This implies that this cut-off is robust independently of the exact shape classification. Ever since, Storch's method is highly recommended for acanthocyte testing and would have served as the control method of choice for the samples analyzed by Yu et al. It should also be mentioned that among the five HD patients of Storch et al. none showed significant acanthocytosis.

On a more general level, the current discussion relates to the various obstacles encountered when acanthocyte determinations are performed for clinical diagnosis. The respective approaches are heterogeneous and cut-off values are not universally agreed upon. More appropriate methods such as Storch et al.'s or SEM, along with respective expertise, are not available everywhere. Artifacts (resulting from technique and human factors in shape classification) seem common and can lead to both false positive and false negative results. Furthermore, in a single patient the number of acanthocytes can vary over time, including total absence (Malandrini et al., 1993; Sorrentino et al., 1999; Bayreuther et al., 2010).

The use of acanthocytosis as diagnostic tool in huntingtonism clearly needs great caution and should be supported by the clinical context and other laboratory findings including elevation of creatine kinase and liver enzymes. Decreased erythrocyte sedimentation rate (ESR) emerges as a much simpler indirect indicator of acanthocytosis and may be a possible diagnostic biomarker (Darras et al., 2021; Rabe et al., 2021).

To sum up, we strongly doubt that the authors' conclusion is warranted, and suspect that it only serves to contribute to confusion in the literature. It would have required appropriate controls, i.e., use of alternative methods such as the wet blood smear technique (Storch et al., 2005), the use of artificial networks for discrimination of acanthocytes from other abnormally shaped erythrocytes (Simionato et al., 2021), or proxy ESR measurements (Darras et al., 2021). This discussion highlights the phenotypic complexity of the neurodegenerative/neurogenetic disorders characterized by chorea.

## Author contributions

All authors listed have made a substantial, direct, and intellectual contribution to the work and approved it for publication.

## Funding

KP was supported by the Rostock Academy of Science (RAS).

## Conflict of interest

The authors declare that the research was conducted in the absence of any commercial or financial relationships that could be construed as a potential conflict of interest.

## Publisher's note

All claims expressed in this article are solely those of the authors and do not necessarily represent those of their affiliated organizations, or those of the publisher, the editors and the reviewers. Any product that may be evaluated in this article, or claim that may be made by its manufacturer, is not guaranteed or endorsed by the publisher.
